# Resection of a large esophageal schwannoma: operative transition from submucosal tunneling endoscopic resection to partial full-thickness resection

**DOI:** 10.1055/a-2418-0562

**Published:** 2024-10-16

**Authors:** Yipeng Yuan, Jixin Zhang, Long Rong

**Affiliations:** 1 26447 Department of Endoscopy Center, Peking University First Hospital, Beijing, China; 2 26447 Department of Pathology, Peking University First Hospital, Beijing, China

The vast majority of esophageal tumors are malignant esophageal cancers, with benign esophageal tumors accounting for a negligible fraction, approximately less than 1% according to previous limited autopsy studies 1 . Primary schwannoma in the digestive tract is rare, especially in the esophagus, which is easily misdiagnosed as leiomyoma.

We report here the case of a 62-year-old patient referred to our unit complaining of acid regurgitation, belching, and postprandial abdominal distension. Gastroscopy showed a hemispherical lesion 22–25 cm from the incisor with smooth surface mucosa, measuring 4 × 4 cm ( Fig. 1 ). Endoscopic ultrasonography showed that the tumor originated from the muscularis propria layer, was mixed hypoechoic, with a hard texture and no blood flow signal.

**Fig. 1:**
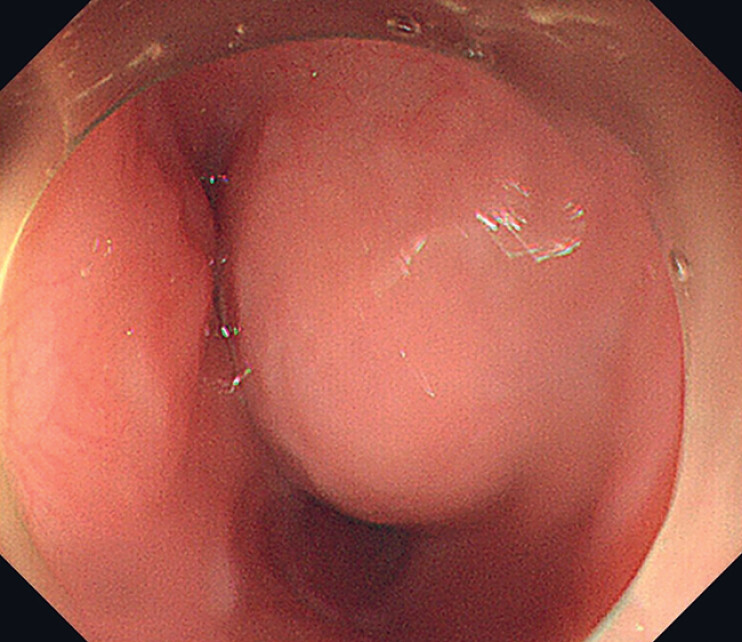
A hemispherical lesion 22–25 cm from the incisor with smooth surface mucosa in white light endoscopy.

A preoperative diagnosis of the esophageal mass was made, and it was decided to perform submucosal tunneling endoscopic resection (STER) ( Video 1 ). However, due to the large tumor, abundant local blood supply, and insufficient tunneling operation space during the procedure ( Fig. 2 ), the mucosal layer was incised and endoscopic submucosal excavation was converted to provide a larger operation space and a clearer operation field. Because the tumor was partially located in the deep muscularis propria, a partial full-thickness resection was performed ( Fig. 3 ).

Resection of a large esophageal schwannoma: operative transition from submucosal tunneling endoscopic resection to partial full-thickness resection.Video 1

**Fig. 2:**
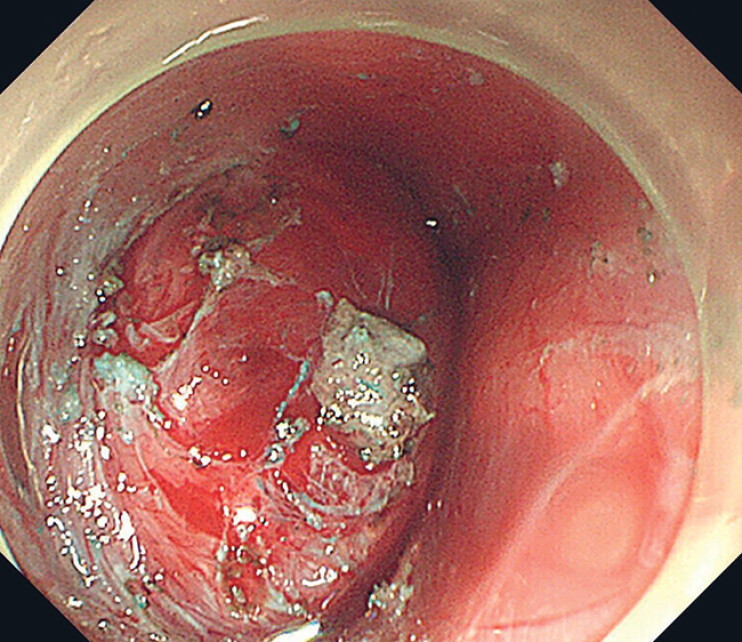
The tumor in the tunnel space with abundant local blood supply.

**Fig. 3:**
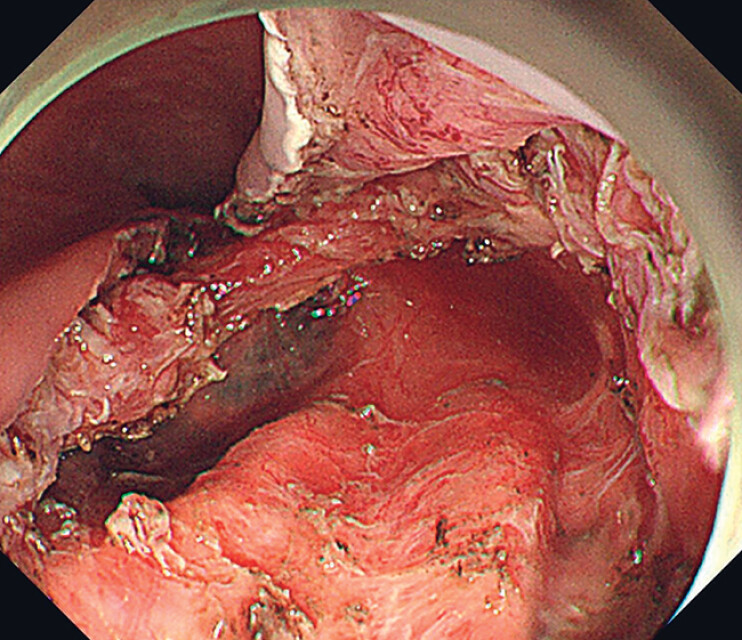
The post-operative wound and partially visible pleura after partial full-thickness resection.

The tumor was completely removed ( Fig. 4 ) but caused partial mucosal tearing when removed from the esophagus because of the size. Histology revealed a schwannoma with S-100 and SOX10 positive, Desmin, and CD34 negative ( Fig. 5 ).

**Fig. 4:**
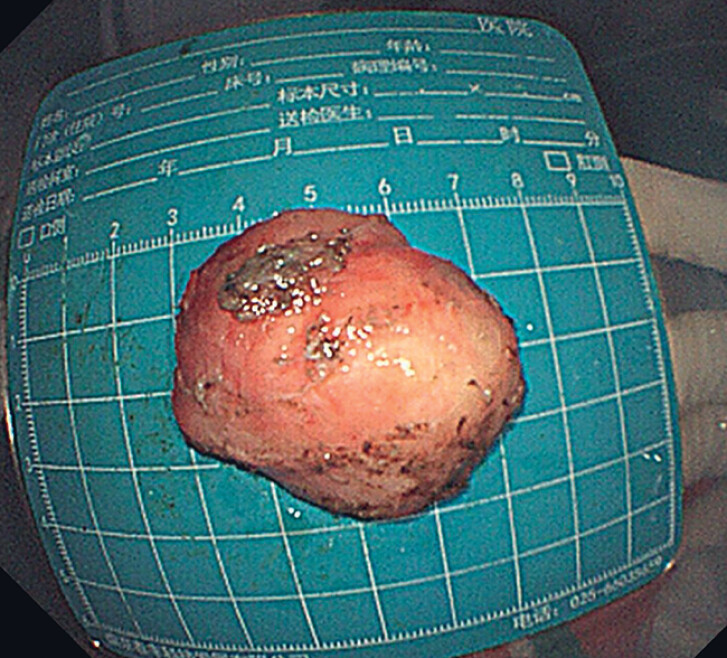
The completely removed tumor with an axial cross-section of about 4 × 4 cm.

**Fig. 5:**
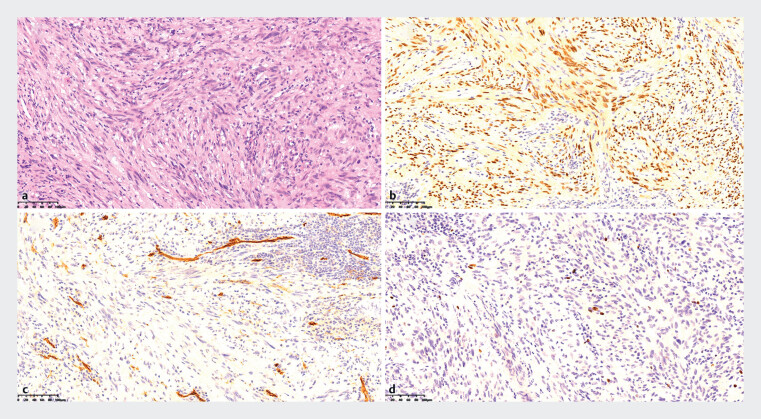
Histology and Immunohistochemical staining of the tumor. **a** Histological examination of the tumor showed spindle-shaped cells arranged in the shape of swirls, bundles and fences. **b** SOX10 positive cells. **c** CD34 negative cells. **d** The mitotic activity rate was 5% on Ki-67 staining.

The indications for endoscopic resection of esophageal submucosal tumors are still controversial 2 3 . In this case, the tumor was ellipsoidal, with an axial cross-section of about 4 × 4 cm, occupying a large space in the tunnel cavity. Complete removal of the tumor was difficult with STER, but the dissection in the tunnel space could still reveal the tumor well. It is necessary to convert the operative method according to the specific situation. Further studies are needed to clarify the indications for STER.

Endoscopy_UCTN_Code_TTT_1AO_2AG_3AZ

## Notes

Endoscopy E-Videos https://eref.thieme.de/e-videos
*E-Videos* is an open access online section of the journal *Endoscopy* , reporting on interesting cases and new techniques in gastroenterological endoscopy. All papers include a high-quality video and are published with a Creative Commons CC-BY license. Endoscopy E-Videos qualify for HINARI discounts and waivers and eligibility is automatically checked during the submission process. We grant 100% waivers to articles whose corresponding authors are based in Group A countries and 50% waivers to those who are based in Group B countries as classified by Research4Life (see: https://www.research4life.org/access/eligibility/ ). This section has its own submission website at https://mc.manuscriptcentral.com/e-videos .
